# Visualizing and quantifying ^33^P uptake and translocation by maize plants grown in soil

**DOI:** 10.3389/fpls.2024.1376613

**Published:** 2024-06-14

**Authors:** Maire Holz, Eva Mundschenk, Valerie Pusch, Rainer Remus, Maren Dubbert, Eva Oburger, Christiana Staudinger, Matthias Wissuwa, Mohsen Zarebanadkouki

**Affiliations:** ^1^ Research Area Landscape Functioning, Leibniz Centre for Agricultural Landscape Research (ZALF), Müncheberg, Germany; ^2^ Department of Forest and Soil Sciences, Institute of Soil Research, University of Natural Resources and Life Sciences (BOKU), Vienna, Austria; ^3^ PhenoRob Cluster of Excellence and Institute of Crop Science and Resource Conservation (INRES), University of Bonn, Bonn, Germany; ^4^ Professorship for Soil Biophysics and Environmental Systems, TUM School of Life Sciences, Technical University of Munich (TUM), Munich, Germany

**Keywords:** ^33^P-imaging, maize (Zea mays L.), root tip, phosphate nutrition, phloem transport

## Abstract

Phosphorus (P) availability severely limits plant growth due to its immobility and inaccessibility in soils. Yet, visualization and measurements of P uptake from different root types or regions in soil are methodologically challenging. Here, we explored the potential of phosphor imaging combined with local injection of radioactive ^33^P to quantitatively visualize P uptake and translocation along roots of maize grown in soils. Rhizoboxes (20 × 40 × 1 cm) were filled with sandy field soil or quartz sand, with one maize plant per box. Soil compartments were created using a gravel layer to restrict P transfer. After 2 weeks, a compartment with the tip region of a seminal root was labeled with a NaH_2_^33^PO4 solution containing 12 MBq of ^33^P. Phosphor imaging captured root P distribution at 45 min, 90 min, 135 min, 180 min, and 24 h post-labeling. After harvest, ^33^P levels in roots and shoots were quantified. ^33^P uptake exhibited a 50% increase in quartz sand compared to sandy soil, likely attributed to higher P adsorption to the sandy soil matrix than to quartz sand. Notably, only 60% of the absorbed ^33^P was translocated to the shoot, with the remaining 40% directed to growing root tips of lateral or seminal roots. Phosphor imaging unveiled a continuous rise in ^33^P signal in the labeled seminal root from immediate post-labeling until 24 h after labeling. The highest ^33^P activities were concentrated just above the labeled compartment, diminishing in locations farther away. Emerging laterals from the labeled root served as strong sinks for ^33^P, while a portion was also transported to other seminal roots. Our study quantitatively visualized ^33^P uptake and translocation dynamics, facilitating future investigations into diverse root regions/types and varying plant growth conditions. This improves our understanding of the significance of different P sources for plant nutrition and potentially enhances models of plant P uptake.

## Introduction

Root traits are of paramount importance for plant P uptake as they significantly influence the plant’s ability to acquire P from soil. Some aspects of root traits, such as root architecture and its impact on P uptake, as well as the genetics of P transporters are relatively well-understood (Hinsinger et al., 2011; Niu et al., 2013; Péret et al., 2014). In contrast, to date, it is still poorly understood how different root types and root segments contribute to P uptake. The contribution of different root sections to P uptake in annual plants has been a subject of research, with two proposed patterns of absorption. Some studies (Burley et al., 1970; Rovira and Bowen, 1970; Rubio et al., 2004) suggest that P is absorbed relatively evenly across the entire root axis. In contrast, other studies (Bowen and Rovira, 1967; Clarkson et al., 1978; Ernst et al., 1989) indicate that P is most actively taken up in the apical regions of the roots.

The aforementioned studies focused on very young plants, quantifying P uptake from seminal or primary roots. However, root systems of cereals exhibit additional root types, including adventitious roots, and various orders of lateral roots (Lynch and Brown, 2012). Despite this, there is a scarcity of research exploring P uptake from different root types. An insightful contribution comes from the studies by Russell and Sanderson (1967) and Rovira and Bowen (1970), which discovered that the absorption of P was higher in lateral roots compared to the primary roots of 10-day-old barley and 5-day-old wheat plants. Similarly, Cochavi et al. (2020) found that P uptake of tomato was notably higher in second- and first-order lateral roots compared to the primary roots.

Notably, the majority of the referenced research was carried out in hydroponic systems, a methodology justified when exploring kinetic capacities and physiological aspects of root P uptake. However, in soil, nutrients with low mobility and high reactivity, such as P, pose challenges for plants in terms of availability. Plants are able to enhance access to limited nutrients, for example, by root exudation or enhanced growth of lateral roots or root hairs (Dakora and Phillips, 2002; Vives-Peris et al., 2020). In order to address the interactions between plants and soil, it is important to employ methodologies that enable the precise determination of P uptake from different soil types and across various root types and regions.

In comparison to studies focusing on P uptake, research addressing the translocation of P within the root system has been relatively limited. Some studies have quantified P translocation from specific root regions or types to the shoot (Russell and Sanderson, 1967; Burley et al., 1970; Clarkson et al., 1978) or quantified the amount of P transported away from specific root sections after uptake (Rovira and Bowen, 1970). However, to the best of our knowledge, there has been no attempt to visually and quantitatively analyze the translocation of P to different root types and regions with high spatiotemporal resolution.

To assess root nutrient uptake and translocation with high spatial precision, commonly employed methods include the use of microelectrodes and radioactive tracers (Rubio et al., 2004). Radioactive P tracers such as ^32^P or ^33^P can be combined with phosphor imaging, a 2D method to visualize the distribution of radionuclides in a sample. In contrast to the application of microelectrodes, the application of radioactive (P) tracers is also feasible under unsaturated soil conditions. Rhizobox setups with ³³P-labeled subsoil layers have been used in the past and allowed for the examination of P utilization from deeper soil horizons. Following the respective growth periods, the distribution of ^33^P in both plant and soil was visualized through phosphor imaging (Bauke et al., 2017; Koch et al., 2019; Wolff et al., 2020). While in two instances (Bauke et al., 2017; Koch et al., 2019), images were captured from the intact samples, in Wolff et al. (2020), roots were excised before imaging. However, in all cases, the experimental design lacked consideration for specific root sections responsible for uptake as well as the temporal dynamics of P uptake and translocation patterns within the plant.

The objective of our study was to test a new experimental setup that not only enables visualization and quantification of P uptake from specific root regions of plants grown in soil but that also allows for tracing the P movement within the plant. Specifically, we aimed to visualize and quantify the temporal patterns of ^33^P uptake from young seminal roots and translocation of freshly absorbed P across the entire root system into the shoots over time.

Our experimental setup utilized rhizoboxes filled with either soil or quartz sand. These rhizoboxes were divided into compartments, and thin layers of gravel were employed to prevent nutrient transfer between these compartments. Maize (one plant per rhizobox) was grown, and for each plant, one compartment, where the apical part of one seminal root was present, was labeled with ^33^P tracer. The uptake and translocation of the tracer were then followed with high temporal resolution over a period of 24 h by the application of phosphor imaging. Destructive sampling after 24 h allowed for quantification of ^33^P distribution within the labeled compartment, in the remaining root system as well as in the shoots. While we acknowledge that the rhizobox compartment and ^33^P detection methods themselves are established techniques, the application of a spatial–temporal analysis using this system to track the uptake of P from specific root regions with a relatively high temporal resolution for plants grown in soil is a novel methodological contribution.

## Materials and methods

### Sorption experiment

The soils used for both the sorption and the rhizobox experiment were an organic matter-free quartz sand and a sandy soil collected from a fallow field site on the experimental station of Leibniz-Center for Agricultural Landscape Research (Müncheberg, Germany). Some selected physical and chemical properties of soils are given in **Table 1**. Both the quartz sand and the sandy soil were air-dried and sieved to<2 mm before use.

**Table 1:** Some selected chemical and physical properties of the quartz sand and the sandy soil collected from a field site near Müncheberg, Germany.

	Sand (%)	Silt (%)	Clay (%)	pH (CaCl_2_)	C_org_ (g kg^−1^)	N_tot_ (g kg^−1^)	P_tot_ (mg kg^−1^)
Quartz sand	100	0	0	6.61	0.06	0.06	5
Sandy soil	87	9	4	4.70	3.88	0.39	439

In order to test the fertilizer P sorption of the quartz sand and the sandy soil, a sorption experiment was performed. For this, 2 kBq ^33^P-labeled phosphoric acid (H_3_^33^PO_4_) (Hartmann Analytic GmbH, Brunswick, Germany) was mixed with a high-P (0.03 g P L^-1^) and a low-P (0.0015 g P L^-1^) Na_2_HPO_4_ solution. Then, 500 mg of the sandy soil and the quartz sand was separately mixed with 1,200 µL spiked ^33^P-nutrient solution and shaken for 60 min on a horizontal shaker. The tubes were subsequently centrifuged at 25,830 *g* for 5 min at 4°C. Of each tube, 600 µL of solution was taken from the supernatant and mixed with 5 mL of UltimaGold-Scintillator (PerkinElmer, USA) and ^33^P was quantified using a liquid scintillation counter (LSC; TriCarb 2800 TR, PerkinElmer, Rodgau, Germany). Sorption of ^33^P to the soil was calculated as the amount of P (%) that was not recovered in the solution.

### Rhizobox setup

**Figure 1:**
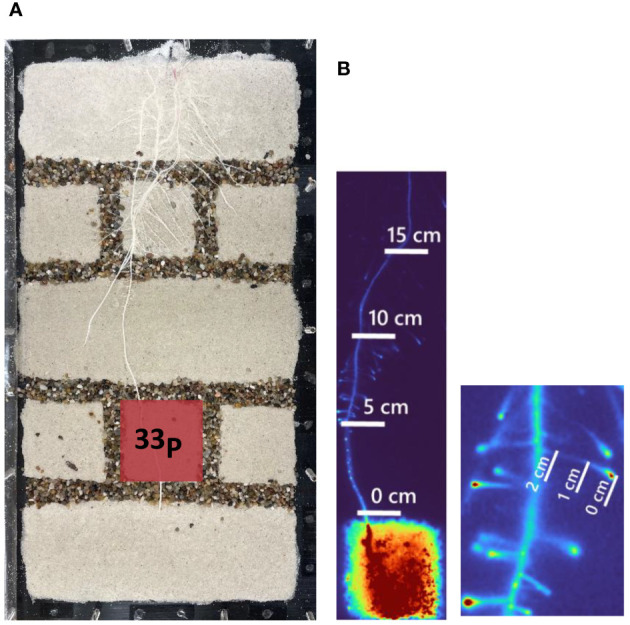
**(A)** Exemplary rhizobox setup that was used for ^33^P labeling. The red square indicates the compartment that was labeled with ^33^P. The inner size of the rhizobox was 20 × 40 cm. Right: **(B)** Exemplary excerpts from the phosphor images, indicating the root segments that were segmented and where ^33^P was quantified. The image on the left side shows the main root that was segmented at four positions, starting from the top of the labeled compartments (0 cm) till 15 cm from the labeled compartment. The lateral roots were segmented in three positions, starting at the root tip (0 cm) till 2 cm from the root tip (right side).

Per soil type (quartz sand and sandy soil), five PVC made rhizoboxes (20 × 40 × 1 cm) were prepared. These rhizoboxes had detachable transparent walls on one side, facilitating uniform soil filling and enabling also monitoring and sampling of roots. The rhizoboxes were positioned horizontally and divided into six small compartments (5.5 × 5.5 cm) and three large compartments (7 × 20 cm). This division was achieved by inserting a PVC frame with a width of 2 cm and a thickness of 1 cm and filling the resulting inner spaces uniformly with respective soil types (sandy field soil or quartz sand). Then, the soil was gently moistened with water by top spraying. Subsequently, the remaining space between soil compartments was filled with a 2-cm gravel layer and the PVC frames were removed. This gravel layer served as a capillary barrier, effectively restricting the movement of P into the targeted soil region (**Figure 1**). Before closing, a thin plastic film was placed between the soil surface and the plastic cover of the rhizobox in order to avoid soil and root disturbance upon opening of rhizoboxes. The rhizoboxes were then positioned at a 55° angle, allowing roots to grow along the transparent wall on the front cover of the rhizoboxes. One pregerminated maize seed (*Zea mays* L.; KWS: *Otto*) was placed at a depth of 1 cm in each rhizobox. During plant growth, the soil moisture was kept at 20%–23% volumetric water content and plants were watered with a half strength Yoshida solution composed of the following (in mmol): 2.86 NH_4_NO_3_, 1 CaCl_2_, 1 MgSO_4_, 1 K_2_SO_4_, and 0.32 NaH_2_PO_4_ × 2 H_2_O and the micronutrients (in µmol): 9 MnCl_2_ * 4H_2_O, 0.5 (NH_4_)_6_Mo_7_O_24_ * H_2_O, 18.5 H_3_BO_3_, 0.16 CuSO_4_ * H_2_O, 36 Fe EDTA, and 0.15 ZnSO_4_ * H_2_O (Yoshida et al., 1976). The temperature in the climate chamber was 20°C during the day and 18°C during the night. The photoperiod was 14 h and the light intensity was 300 µmol m^-2^ s^-1^.

After 10–12 days of plant growth, one small rhizobox compartment containing the root tip region of one seminal root was labeled with 4.32 mL of a 0.15 mM NaH_2_^33^PO_4_ solution. In total, 12 MBq ^33^P was added with the solution per plant. To add the labeling solution, the rhizoboxes were opened and the solution was distributed carefully and evenly over the entire surface of the compartment using a syringe. Phosphor imaging was used to visualize P uptake at 45 min, 90 min, 135 min, 180 min, and 24 h post-labeling. One additional plant was imaged every 45 min over a period of 8 h. Prior to phosphor imaging, a photo was taken from the rhizobox surface. Phosphor imaging screens (Storage phosphor screen, BAS-IP MS 2040; Fujifilm, Japan) were carefully placed on the rhizobox surface with an exposure time of 45 min. The imaging screens were scanned in the dark using a CR-Scanner (HD-CR 35 NDT, Dürr NDT GmbH & Co. KG, Germany) at a resolution of 50 µm.

### Sample preparation and analysis

After the last imaging, shoots were cut and removed directly above the soil surface and dried at 60°C for 48 h. After shoot harvest, the labeled seminal root including its laterals was separated from the root system and taken out from the soil. While removing it from the soil, it was divided in the following root sections: (a) the root section below the labeled compartment, (b) the root section within the labeled compartment, and (c) the root section above the labeled compartment that included its lateral roots. Thereafter, the remaining root system was taken out of the soil. Soil adhering to the roots was removed and the root sections and the remaining root system were dried at 60°C for 48 h. After drying, the shoot biomass and the biomass of the root sections were determined. The root pieces from each section were then visualized by phosphor imaging as described above. Thereafter, all samples were ground to a fine powder using a high-speed ball mill (Retsch M 400, Haan, Germany). For radioactive P analysis, shoot and root material was pressure digested in 64% HNO_3_ (König, 2005). The resulting solution was mixed with 10 mL of scintillation cocktail (Rotiszint eco high, Roth, Karlsruhe, Germany) and ^33^P using liquid scintillation counting (LSC) (Tri-Carb^®^ 2800TR, Perkin Elmer, Germany). To test the reliability of the imaging approach, we calculated the relation between the gray values obtained from imaging of the excavated root sections and the quantified ^33^P activity of the excavated root sections (**Supplementary Figure S1**).

### Image calibration and analysis

The digital images obtained during phosphor imaging were further processed in Matlab R2023a (The MathWorks Inc., USA). To account for the impact of radioactive decay on signal intensity (*t*_1/2_ of ^33^P is 25.3 days), we first applied a radioactive decay law to all images to recalculate the ^33^P to the start of the labeling experiment. Then, the images obtained from each rhizobox at discrete time intervals underwent image registration processes to ensure alignment with a predefined reference position (image taken at 24 h after labeling). To achieve this, an intensity-based registration technique in Matlab incorporating a rigid geometrical transformation algorithm, allowing for *x* and *y* translation, as well as rotation, was implemented. This algorithm facilitated the alignment of images, compensating for spatial discrepancies and ensuring accurate overlay for subsequent analyses. In each image, a soil region free of ^33^P was selected, and its gray value was subtracted from the root regions to be analyzed to normalize the captured signal between all images and remove any variation in background signal among different images.

To quantify the ^33^P uptake and translocation, we created a mask image that highlights some selected seminal and lateral roots transporting ^33^P beyond the labeled compartment. This procedure was conducted using paint.net, utilizing thresholding methods applied to the histogram of the image captured at the end of the experiment. Following this initial step, we manually refined the segmented regions through visual inspection, aiming for precise identification of the targeted positions of seminal and lateral roots. To enhance the accuracy of our quantification and mitigate uncertainties arising from potential overlapping root segments, we decided to selectively choose root segments [regions of interest (ROIs)] with a length of 0.5 cm at intervals of 5 cm along the seminal root, starting right above the injected compartment. (i.e., 0 cm) and ending 15 cm above the labeled compartment (**Figure 1**). Similarly, for lateral roots, we selected three ROIs with length of 0.2 cm at intervals of 1 cm (**Figure 1**). All captured images were multiplied by this mask and then captured gray values along segmented roots were calculated in Matlab.

For the phosphor images from the excavated root sections, the background signal was removed and the gray values of each root section were summed up in order to correlate the gray values to the ^33^P activity in the root sections that were determined by LSC.

For calibration, we prepared 0.15 mM NaH_2_^33^PO_4_ solutions with increasing ^33^P activities: 0.00, 0.84, 1.67, 3.34, 6.69, 13.37, and 26.75 kBq cm^-2^. Pieces of filter paper (4 × 4 cm) were cut, soaked with 68.5 µL of the solution, and imaged as described for phosphor imaging. To obtain the calibration function, the ^33^P activities per cm^2^ were converted to ^33^P activity per pixel (Bq px^-1^). Consistent with the steps applied to phosphor images of roots, we calculated the gray value captured on the control filter paper where no ^33^P activity was added and subtracted it from all pixels. The ^33^P activities were then related to the gray values of the images. An equation was fitted through the data of gray value and ^33^P activity to obtain a calibration function to convert the gray value to ^33^P activity in Bq px^-1^.

The root surface area of the seminal root in the labeled compartment was calculated using Fiji (1.6.0) (Lobet et al., 2011) based on the length of the root within the compartment and the root diameter.

### Calculations and statistics

To account for the decay time between the setup of experiments and the measurement of ^33^P activity, data were corrected for the ^33^P half-life (*t*_1/2_) of 25.34 days using the following Equation 1:


(1) $$\text{N}\left({\text{t}}_{1}\right)\;={\text{N}}_{0}*\text{ e}\hat{\;}\left(-\lambda \text{t}\right)$$

where *N*(*t*) is the activity of the sample at time *t*_1_ in kBq, *N*_0_ is the initial activity of the sample in kBq (at time *t* = 0), *λ* is the decay constant, specific to the isotope, and determines the rate of decay, and *t* is time in hours.

The amount of P derived from fertilizer was calculated using Equation 2:


(2) $$\text{P derived from fertilizer }\left[{\text{mg P plant}}^{-1}\right]\;=\;\frac{{\;}^{33}\text{P uptake plant }\left[{\text{kBq plant}}^{-1}\right]}{\text{S.A of labeled fertilizer }\left[{\;}^{33}{\text{P}(\text{ kBq mg}}^{-1}\;P\right]}$$

where S.A. of labeled fertilizer is the specific activity of labeled fertilizer expressed as ^33^P activity per mg P fertilizer.

For the statistical data analysis, the software R (version 4.1.3, R Core Team (2023)) and the R packages nlme and ggplot were used. The data on plant biomass and fertilizer-derived P uptake were not normally distributed based on results of the Shapiro–Wilk and based on visual inspection of the qq plots. The data were therefore log-transformed, which resulted in normal distribution of the data.

For the plant biomass data, the data on fertilizer-derived P uptake of the shoot, and the whole root system and for the P uptake per cm^-2^, a *t*-test (*p*< 0.05) was conducted to test for significant differences between the two soil types.

To test for significant differences in fertilizer P recovery in several root sections, a linear mixed-effect model was used. The model included soil type (quartz sand and sandy soil) and root section (below compartment, within compartment, above compartment, and remaining roots) as fixed factors and plant number (i.e., replicates 1–5) as a random factor to account for the fact that measurements on different root sections were conducted on the same plants (i.e., repeated measurements). Following the model, a *post-hoc* test (Tukey HSD) was conducted to test for significant differences between factor levels. The level of significance was *p*< 0.05.

## Results

Plant grown in the sandy soil showed higher shoot biomass (184.5 mg plant^−1^) than those grown in quartz sand (105.5 mg plant^−1^) while the root biomass did not differ significantly between the two soils (sandy soil: 156 mg plant^−1^; quartz sand: 248.5 mg plant^−1^) (**Table 2**). In total, plants grown in quartz sand allocated three times more P from fertilizer into their shoots and roots compared to the plants grown in sandy soil. Accordingly, the difference in P uptake per cm^2^ root surface was in a similar range and was found to be 2.4 times higher in the quartz sand compared to the sandy soil (**Table 2**).

**Table 2:** Plant biomass, P derived from fertilizer in shoot and root, ^33^P recovery in shoot and root, and P uptake per root surface for plants grown either in quartz sand or in sandy soil.

	Biomass (mg)		P derived from fertilizer (µg plant^−1^)		^33^P recovery (%)		P uptake from fertilizer (µg P cm^-2^ root surface d^-1^)
	Shoot	Root	Shoot	Root	Shoot	Root	
Quartz sand	105.52^a^ (27.41)	248.56^a^ (25.83)	0.15^b^ (0.04)	0.18^b^ (0.04)	0.79^a^ (0.23)	0.93^a^ (0.21)	0.15^b^ (0.03)
Sandy soil	184.47^b^ (38.17)	175.97^a^ (18.60)	0.05^a^ (0.01)	0.05^a^ (0.01)	0.32^b^ (0.15)	0.29^b^ (0.21)	0.06^a^ (0.01)

Variation is given as standard error. The small letters indicate significant differences between the two soil types based on a t-test (p< 0.05).

**Figure 2:**
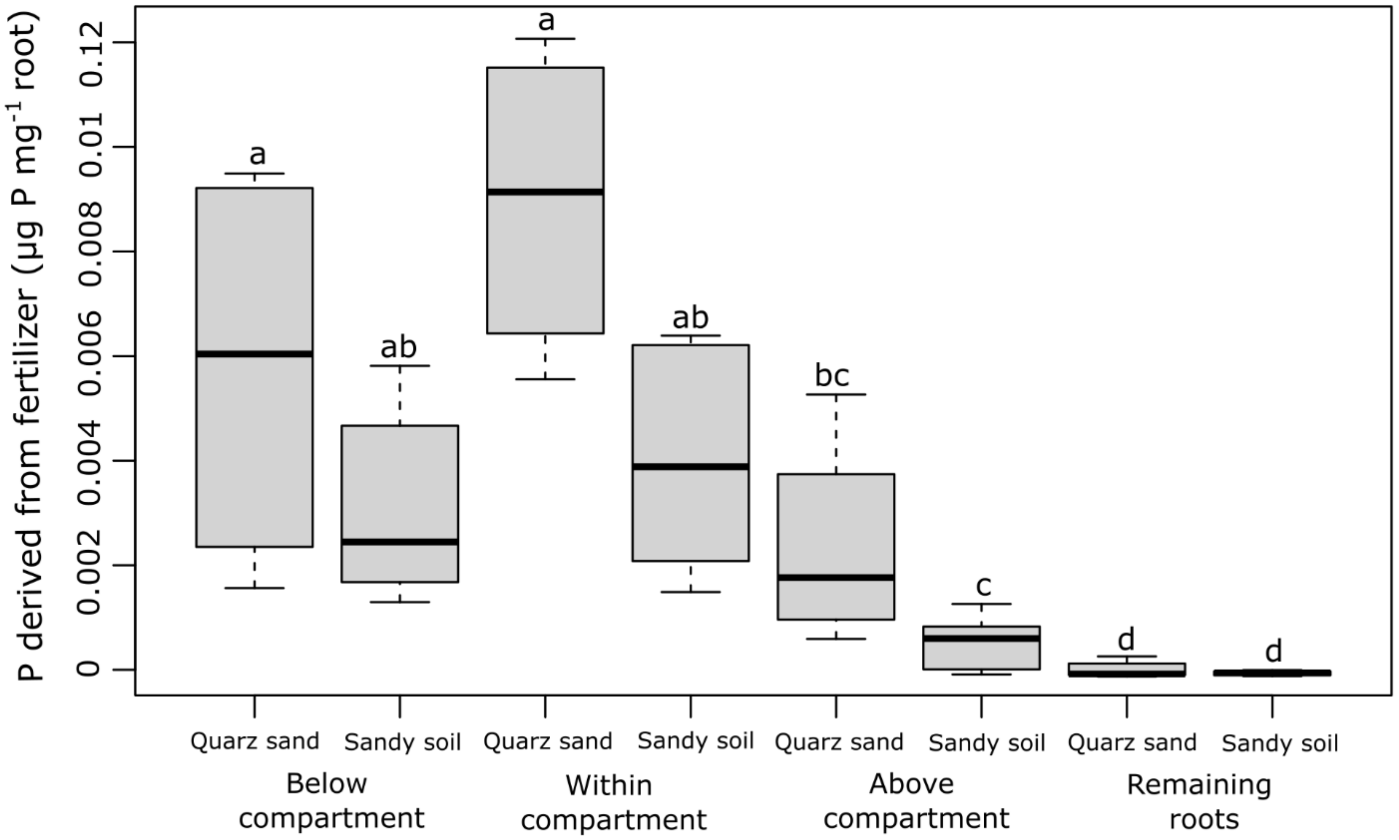
Concentration of P derived from fertilizer (µg P mg^−1^ root) for each sampled root section (the root section below the labeled compartment, the root section within the labeled compartment, the root section above the labeled compartments, and the remaining root system) and both soil types. The black lines in the boxes indicate the median. The lower and upper hinges correspond to the first and third quartiles (the 25th and 75th percentiles) while the upper and lower whiskers extend from the hinge to the largest and the smallest values, limited to a maximum of 1.5 times the interquartile range. The small letters above the boxes indicate statistical differences based on the mixed-effect model after conducting a Tukey-HSD test (*p*< 0.05).

**Figure 3:**
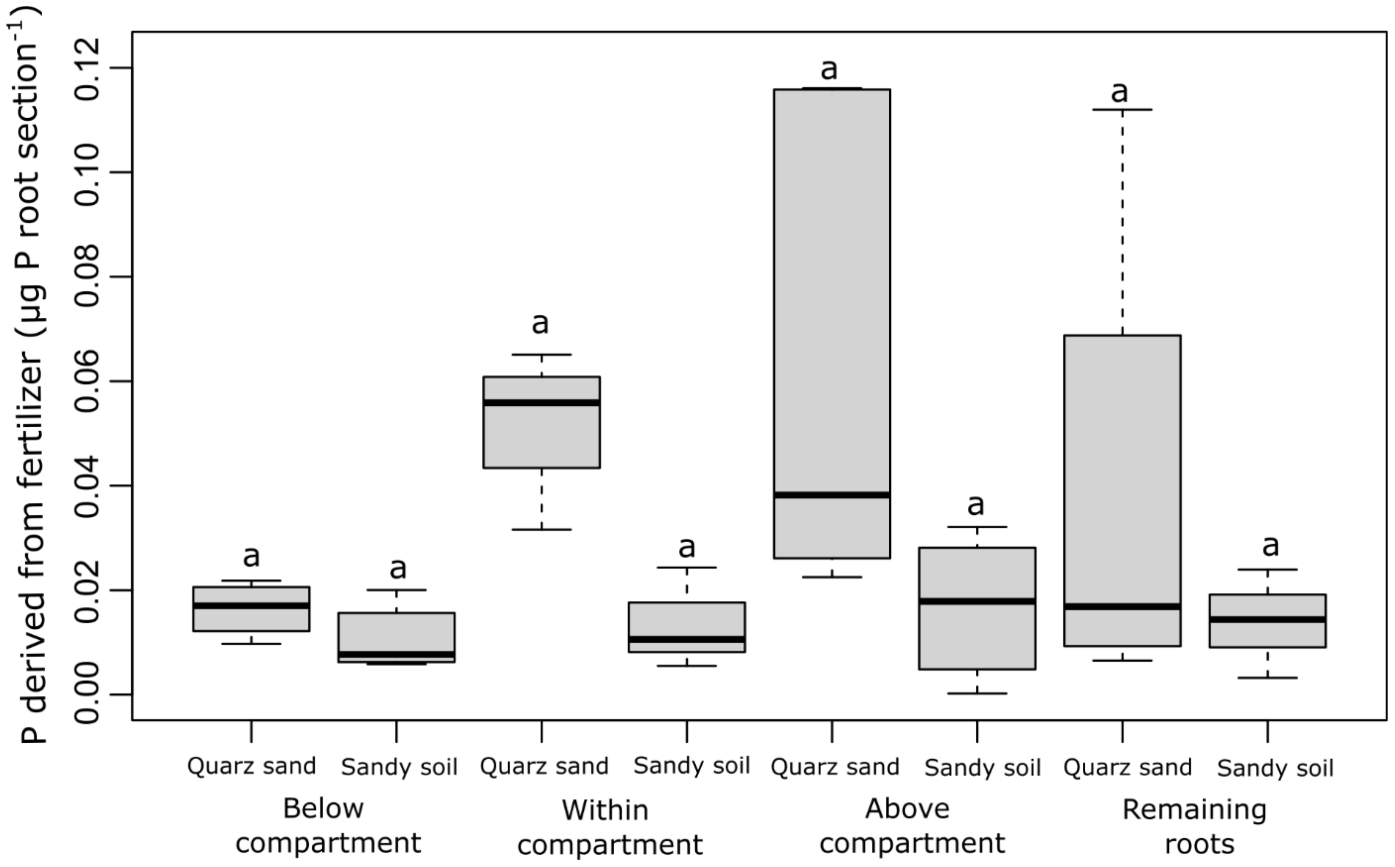
Content of P derived from fertilizer (µg P root section^-1^) for each sampled root section (the root section below the labeled compartment, the root section within the labeled compartment, the root section above the labeled compartments, and the remaining root system) and both soil types. The black lines in the boxes indicate the median. The lower and upper hinges correspond to the first and third quartiles (the 25th and 75th percentiles) while the upper and lower whiskers extend from the hinge to the largest and the smallest values, limited to a maximum of 1.5 times the interquartile range. The small letters above the boxes indicate statistical differences based on the mixed-effect model after conducting a Tukey-HSD test (*p*< 0.05).

The P concentration derived from fertilizer (µg P mg^−1^ root) strongly differed between the different root sections that were sampled (the root section below the labeled compartment, the root section within the labeled compartment, the root section above the labeled compartments, and the remaining root system) but did not differ between the soil types (**Figure 2**). With 0.004–0.009 µg P mg^−1^ root, the highest fertilizer-derived P concentrations were found in the roots growing inside and below the labeled compartments, i.e., in the root tip region of the labeled root and the region behind the root tip. In the root section directly above the labeled compartment, on average 5 times lower P concentrations were observed (0.0007–0.002 µg P mg^−1^ root), while in the remaining root system, fertilizer-derived P was approximately 50 times lower than in the root sections within or below the labeled compartment (**Figure 2**). In contrast to the P concentrations in the root sections, the fertilizer-derived P content (µg P root^−1^) differed neither between the root sections nor between the soil types (**Figure 3**). The values ranged between 0.01 and 0.05 (µg P root^−1^) with no significant differences (*p* > 0.05).

**Figure 4:**
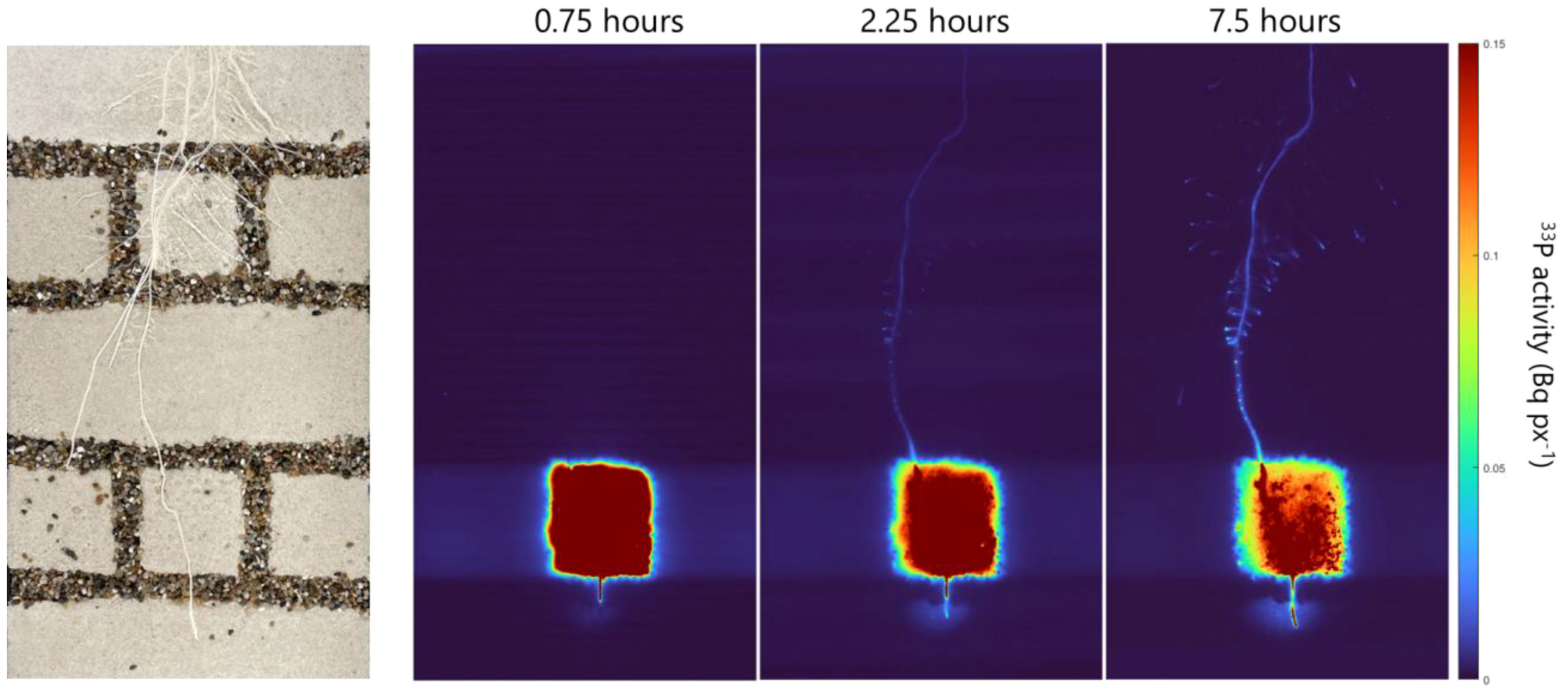
Exemplary images of the soil–root interface of one rhizobox (left) and the corresponding phosphor images exemplary shown for the time points of 0.75, 2.25, and 7.5 h after labeling.

By labeling one soil compartment with 12 MBq of ^33^P, we achieved a strong and homogeneous ^33^P signal within the compartment (**Figure 4**; **Supplementary Video S1**). After 1.5 h, a low ^33^P signal was seen in the whole lengths of the labeled root and this signal intensified within the 24 h of imaging (**Supplementary Video S1**). While the strongest ^33^P signal accumulated in the root tips of the labeled seminal root and its lateral branches (**Figure 4**; **Supplementary Video S1**), faint signals of ^33^P were also detectable in root tips of unlabeled seminal roots and their lateral branches (**Supplementary Figure S2**). The labeling of 12 MBq resulted in a detectable change in ^33^P activity within the root system, which enabled us to select several root regions and to quantify the change in activity in these root regions.

**Figure 5:**
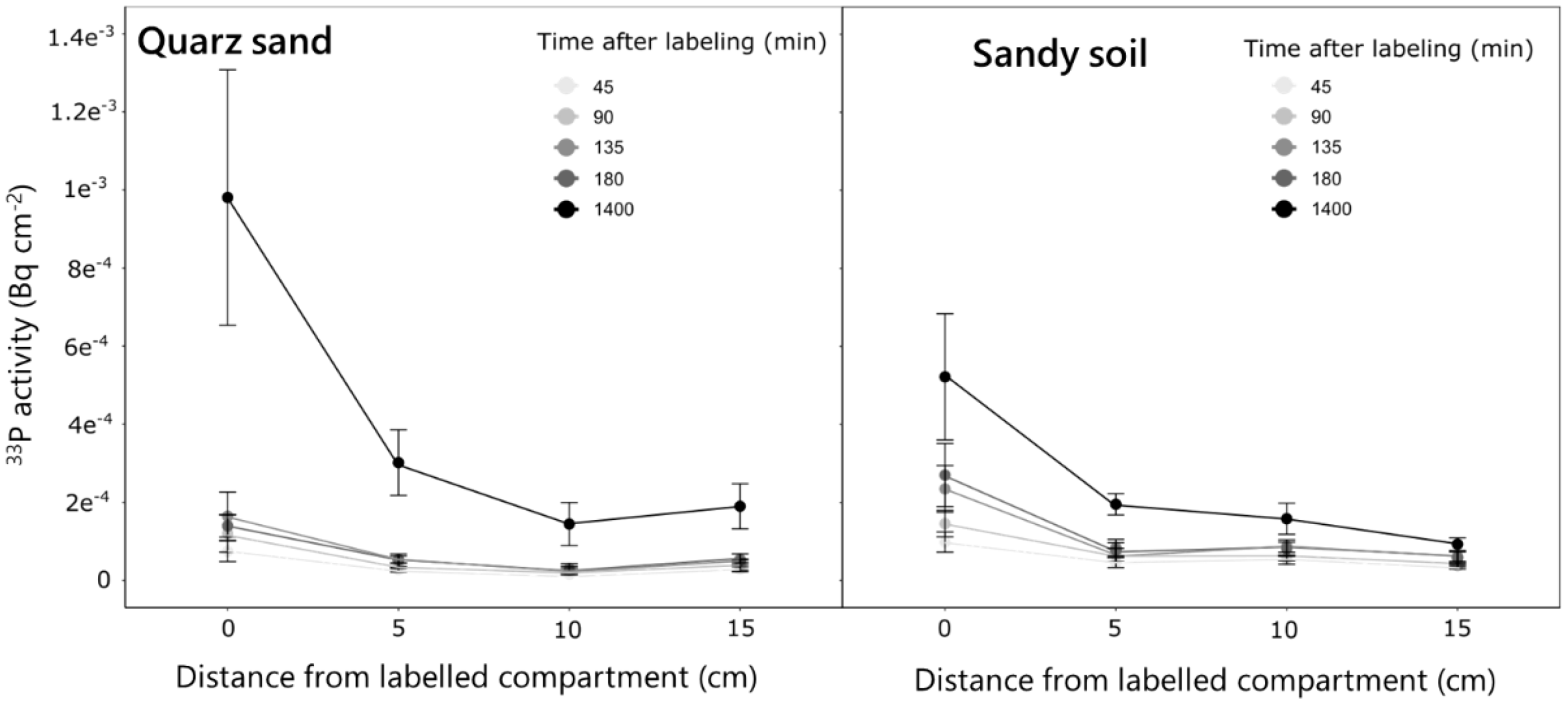
Change in ^33^P activity over time in the ROIs of the labeled seminal roots of plants grown in the quartz sand and the sandy soil. Measurements were done along the labeled seminal root in the four selected locations over time for five replicate rhizoboxes per soil type. Variation is given as standard error.

**Figure 6:**
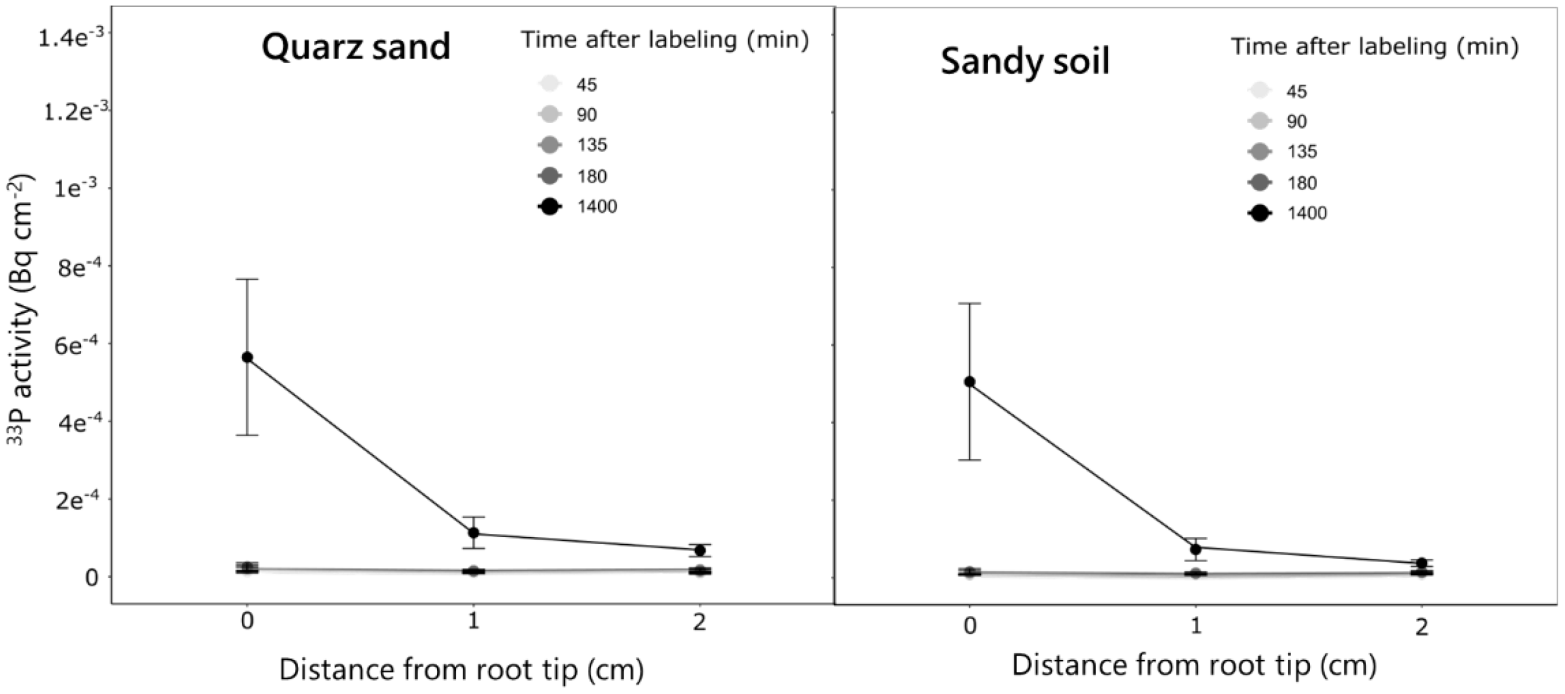
Change in ^33^P activity over time in the ROIs of the lateral roots emerging from the labeled seminal root for plants grown in the quartz sand and the sandy soil. Measurements were done at three positions starting at the root tip at lateral roots emerging from the labeled seminal roots over time. The analysis was done for five replicate rhizoboxes per soil type from which each three lateral roots were selected. Variation is given as standard error.

The experiment revealed distinct P uptake and translocation patterns in different soil types. For both quartz sand and sandy soil the strongest accumulation of ^33^P activity in the root occurred closest to the labeled compartment (i.e., 0 cm) and decreased towards the parts closer to the shoots (15 cm) (**Figure 5**). Over time, this increase was more gradual for the sandy soil, while in the quartz sand, the strongest increase in activity was observed between 180 and 1,400 min after labeling. This resulted in an approximately two times higher ^33^P activity in the ROI closest to the labeled compartment (0 cm) 1,400 min after labeling for the quartz sand compared to the sandy soil (**Figure 5**). In contrast to the seminal roots, for lateral roots, no differences in P activity between the two soil types was observed. For both soils, a ^33^P activity of approximately 5^−04^ Bq was observed for the root tip region 1,400 min after labeling (**Figure 6**). The regions 1 and 2 cm behind the root tip showed only slight increases in ^33^P activity after 1,400 min. No change in ^33^P activity within lateral roots was observed for the measurement times of 45–180 min after labeling.

**Figure 7:**
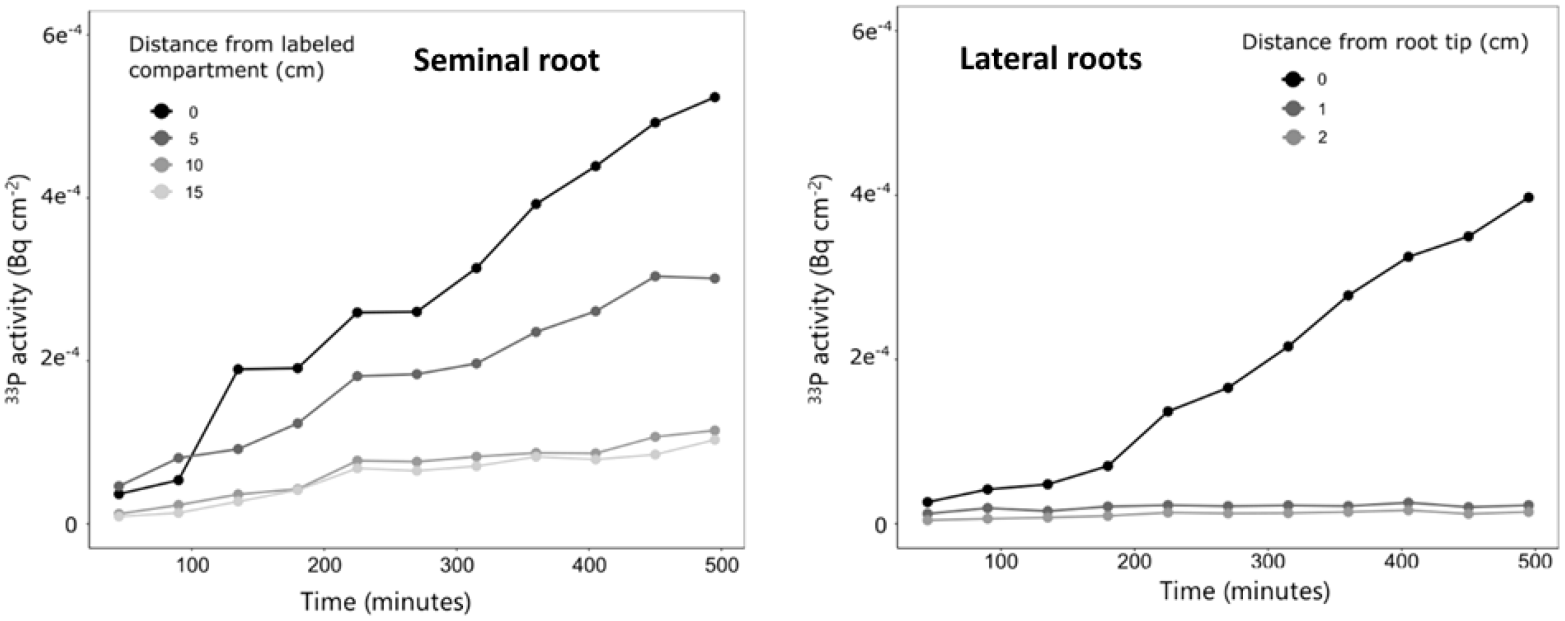
Change in ^33^P activity over time for one plant grown in quartz sand and imaged with a higher temporal resolution than the remaining plants. Measurements were done along the labeled seminal root (left) and along lateral roots (right). The measurements correspond to only one plant and therefore no variation is shown.

For one plant grown in quartz sand, we conducted measurements with a higher temporal resolution, and this plant was imaged over a period of 8 h every 45 min (**Figure 7**). The measurements showed a continuous increase in ^33^P activity over time in all ROIs; however, the increase was strongest in the ROI closest to the labeled compartment (0 cm), followed by the ROI of 5 cm, while at 10 and 15 cm, only a marginal increase in ^33^P activity was observed (**Figure 7**). For the lateral roots of this plant, the ^33^P activity did not change in the regions behind the root tip (ROI of 1 and 2 cm). The ^33^P activity in the root tip started to increase after 180 min, and from there, it continuously increased until the end of the measurement at 8 h after labeling.

To assess the imaging approach’s reliability, we determined the correlation between gray values from imaging excavated root sections and the quantified ^33^P activity of those sections. This relation, though positive and significant, was determined by a few extreme values while most values were found in the lower range of ^33^P activities (**Supplementary Figure S1**).

## Discussion

We demonstrated the effective application of phosphor imaging for precise spatiotemporal assessment of ^33^P uptake and translocation in our study. The tested experimental setup not only allows for high-resolution imaging but also presents an opportunity to explore rhizosphere interactions of soil grown plants. Moreover, it has the potential to establish connections between P uptake from specific root regions or types and P mobilization facilitated by root exudation processes.

Here, we used relatively high specific ^33^P activities of 280 kBq g^−1^ soil for labeling to be able to reduce the incubation times of the imaging screens to 45 min and therefore to increase the temporal resolution of image sequences. For the tested soils, activities were sufficient, and already after 45 min, an increase in ^33^P activity was detected in the labeled roots (**Figures 5**, **7**). However, in soils with higher P sorption than our soils, P uptake by plants might be restricted by stronger sorption. Consequently, even higher specific ^33^P activities in the labeled compartments might be advisable for strongly P sorbing soils. In contrast, in studies where the emphasis lies on spatial rather than temporal resolution and where the imaging plant is positioned on the soil before measurements for several hours, lower specific activities of 5 to 20 kBq g^−1^ are generally sufficient (Bauke et al., 2017; Koch et al., 2019).

It is crucial to acknowledge that phosphor imaging is semi-quantitative. Visualizing the distribution of ^33^P may not provide a quantitative assessment, but it offers a significant advantage by allowing us to monitor the redistribution of tracer P in the soil and in the plant (Bauke et al., 2017). The intensity of radioactive emissions captured by image plates is greatly influenced by the distance between the sample and the screen and possibly by soil covering, for example, ^33^P-labeled roots, as well as by the density of the radioactive sample, which affects the self-attenuation of the sample (Bauke et al., 2017; Holz et al., 2019). Bauke et al. (2017) observed discrepancies in measurements of ^33^P activities in root tips between phosphor imaging and the quantitative determination of ^33^P contents in root tips (determined by LSC counting after extraction of total P). The authors suggested that root sections with fully developed epidermis might shield ^33^P signals more effectively than root tips with thinner tissue, introducing a potential bias. However, in our study, a relatively good relation between imaged-based ^33^P intensity in the labeled roots and ^33^P contents determined by LSC was observed (**Supplementary Figure S1**). This relation should be treated with caution though as the correlation was strongly influenced by a few extreme values. In conclusion, to enhance the accuracy of data obtained from images, this imaging technique should be complemented with LSC measurements (Bauke et al., 2017; Wolff et al., 2020) as done in our study.

Most imaging studies that previously determined uptake rates from young root sections were conducted in nutrient solution. Therefore, those results can most likely be compared with our results from the quartz sand as this substrate did not interact with the P added as fertilizer as indicated by the negligible P sorption (**Table 3**). Russell et al. (1954) reported a P uptake per plant of 24–111 µg P per day, depending on the nutrient solution P concentrations comparable to ours. While our rates are by far lower, it is important to note that in the study by Russell et al. (1954), the whole root system was labeled and information on the root system size or root surface area that was exposed to the labeled P was not reported. As a result, a meaningful comparison with our results remains challenging. Russell and Sanderson (1967) conducted a quantification of P uptake from 3.5-cm root segments of young barley plants. Their findings revealed that plants absorbed approximately 80 µg of P from these root segments within 1 day. These high uptake rates compared to ours might be explained by the fact that in the study by Russell and Sanderson (1967), the P concentrations in the nutrient solution were 10 times higher than those employed in our experiment. Bowen and Rovira (1967) quantified P uptake from 1-cm root segments of young wheat plants, revealing that approximately 0.8 µg of P was absorbed within a single day. While our experiment yielded a comparable range of P uptake, an accurate comparison would necessitate knowledge of the labeled root surface area reported by Bowen and Rovira (1967). Rubio et al. (2004) quantified P uptake from the first 2 cm starting from the root tip of basal roots of 18-day-old common bean plants in nutrient solution. Depending on the P concentrations added during labeling with ^32^P, the labeled segments took up 0.4–2.2 µg P after 24 h, which corresponds to our results. However, also here, an accurate comparison would necessitate knowledge of the root surface area of the labeled root segment.

**Table 3:** P sorbtion to soil matrix (% of added P added with nutrient solution) for both tested soils and two P concentrations.

	P concentration of added nutrient solution	P sorbed to soil matrix (% of added P added with nutrient solution)
Quartz sand	High	0.196
	Low	1.326
Sandy soil	High	10.253
	Low	50.392

To the best of our knowledge, only two other studies report experimental results based on P imaging with plants grown in soil and quantified the uptake of the added label. Ernst et al. (1989) introduced agar strips (21.5 mm × 3.5 mm) labeled with a 0.4 mM ^32^P solution onto root segments of maize plants grown in soil. After 1 day, 20% of the P within the agar strip had been absorbed by the plant. In contrast, our study recorded the uptake of less than 1% of the added labeled P by the plant. It is essential, however, to consider two key factors: (a) the P within the agar is readily available to the plant and does not interact with the soil matrix, and (b) the small size of the agar strip meant that roots were locally exposed to higher labeled P levels compared to our system. Consequently, it is plausible that the plant takes up a larger proportion of this modest amount of added P. Koch et al. (2019) cultivated plants in rhizoboxes, and a layer of either Ferrasol or Luvisol subsoil within the rhizoboxes was labeled with ^33^P-orthophosphate. After a 14-day incubation period, the recovery of the added P source in the shoots was determined. The findings indicated that only 0.1% of the added P source was recovered in the shoots for the Luvisol treatment, while 0.8% was recovered for the Ferrasol treatment. These recovery rates were notably lower at 0.007% per day for the Luvisol and 0.06% for the Ferrasol, and therefore in contrast to the significantly higher rates observed in our study, which were 0.79% for quartz sand and 0.32% for sandy soil after just 1 day. The disparities in recovery rates between the two studies may be attributed to the substantially higher clay and soil organic matter contents in the soils tested by Koch et al. (2019). This difference could lead to a potentially higher sorption of P to the soil matrix in their soils compared to ours. However, it is essential to interpret the comparison cautiously due to variations in experimental conditions. Notably, the compartment size in Koch et al. (2019) was larger than ours, and crucial details such as root biomass and surface area in the labeled compartment were not reported. Consequently, the calculation of P uptake rates per root biomass or surface area was not feasible, making a precise comparison challenging. Furthermore, differences in transpiration rates resulting from distinct growth conditions are also known to impact plant nutrient uptake, especially in the case of mobile nutrients (Barber, 1962). In the case of poorly mobile P, the role of transpiration in enhancing mass flow towards the root surface may be limited. However, high transpiration rates were associated with plant P accumulation in sandy soils (Cernusak et al., 2011; Matimati et al., 2014). The observed variations in P uptake rates highlight the need for considering key factors in result comparisons. These factors include P source accessibility (agar/solution, vs. soil), soil property differences, and, often unreported, root exposure surface area to the ^33^P label.

We observed an increase in ^33^P signal along the labeled seminal roots over the 24 h in which measurements were done. This indicates that part of the P that was taken up remained in the tissues of labeled seminal roots. The strongest increase in ^33^P signal was observed in the root tips of the labeled seminal roots followed by the regions right above the labeled compartment while the signal was less intense in the more mature root parts. This is indicative of both effective translocation and strong sink activity of the root tips even beyond the labeled compartments. As P is a crucial constituent in cell growth and protein biosynthesis (Clowes, 1958; Silk and Erickson, 1980; Frossard et al., 2011; Veneklaas et al., 2012), it seems reasonable that large amounts of recently taken up P are translocated to growing root tips (Kanno et al., 2013). Plants in our experiment translocated P from the region of uptake to the root tips of the labeled root and to the root tips of other seminal roots (**Supplementary Figure S2**) so that after 24 h, only approximately 50% of the absorbed P was translocated to shoots (**Table 2**). It is known that plants typically reutilize a significant portion, at least 50%, of P from senescing leaves (Aerts, 1996) and that this remobilization of P can serve as a substantial source for growth for example under limited soil P availability (Veneklaas et al., 2012). However, the translocation of P within the root system has been barely studied and suggests that P is transported via the phloem to distant growing root tips (Smith et al., 2003). Our results show a rapid P movement within the root system, suggesting efficient P distribution to actively growing root tips within a root system. When roots encounter soil regions with relatively high P availabilities, they might therefore be able to efficiently supply other root segments, especially root tips, with P. Plants likely remobilize P not only from senescing leaves but also from roots potentially aiding young root growth (Lynch and Ho, 2005).

## Conclusions

We demonstrated the effective application of phosphor imaging for precise spatiotemporal assessment of P uptake and translocation in plant–root systems. While recent studies have highlighted the significance of lateral and crown roots in maize for water uptake (Ahmed et al., 2018), the corresponding insights into P uptake are currently lacking. Focusing on seminal roots only, we could confirm that our experimental setup has the potential to uncover the role of different root types in P uptake and translocation. Moreover, incorporating details about the variability in P uptake across various root types would significantly enhance the accuracy of P uptake modeling. Many existing models do not consider potential variations in P uptake between distinct root types (Hinsinger et al., 2011; Dunbabin et al., 2013; Kuppe et al., 2022) while only some consider differences in P uptake with root age (Schnepf et al., 2012). To advance modeling accuracy, it is crucial to offer a more thorough depiction of the specific uptake surfaces relevant to P uptake (Hinsinger et al., 2011), something that could be achieved with the proposed approach. Lastly, the phosphor imaging setup described here also opens additional avenues for investigating rhizosphere interactions. For instance, integrating ^33^P with ^14^C imaging of root exudates (Holz et al., 2017) could enhance our comprehension of how the mobilization of P through root exudation influences and potentially optimizes subsequent P uptake of specific root regions or root types.

## Data availability statement

The raw data supporting the conclusions of this article will be made available by the authors, without undue reservation.

## Author contributions

MH: Conceptualization, Funding acquisition, Investigation, Writing – original draft. EM: Conceptualization, Investigation, Writing – review & editing. VP: Investigation, Writing – review & editing. RR: Conceptualization, Investigation, Writing – review & editing. MD: Writing – review & editing. EO: Conceptualization, Writing – review & editing, Funding acquisition. CS: Conceptualization, Writing – review & editing. MW: Conceptualization, Writing – review & editing. MZ: Conceptualization, Investigation, Writing – original draft, Writing – review & editing.

## Supplementary material

Supplementary Figure 1 Correlation between gray values that were obtained after imaging the excavated root sections and the ^33^P activity of the respective root sections in kBq per root section.

Supplementary Figure 2 Two exemplary images of one rhizobox (left) and the corresponding phosphor images taken and exemplary shown for the measurement 24 h after labeling. The legend to the right refers to both images, the one taken from quartz sand (left) and the one taken from the sandy soil (right).

Supplementary Video 1 Images sequence over 7.5 h of one rhizobox. Activity is shown in Pq px^-1^.
