# Supplementary material for: Visualizing and quantifying 33P uptake and translocation by maize plants grown in soil
Source: Front Plant Sci. 2024 Jun 14;15:1376613. doi: 10.3389/fpls.2024.1376613 (PMC11211545; doi:10.3389/fpls.2024.1376613)
Supplement: Supplementary Figure 1 — Correlation between gray values that were obtained after imaging the excavated root sections and the 33P activity of the respective root sections in kBq per root section. [file DataSheet_1.pdf]

Supplementary Figures

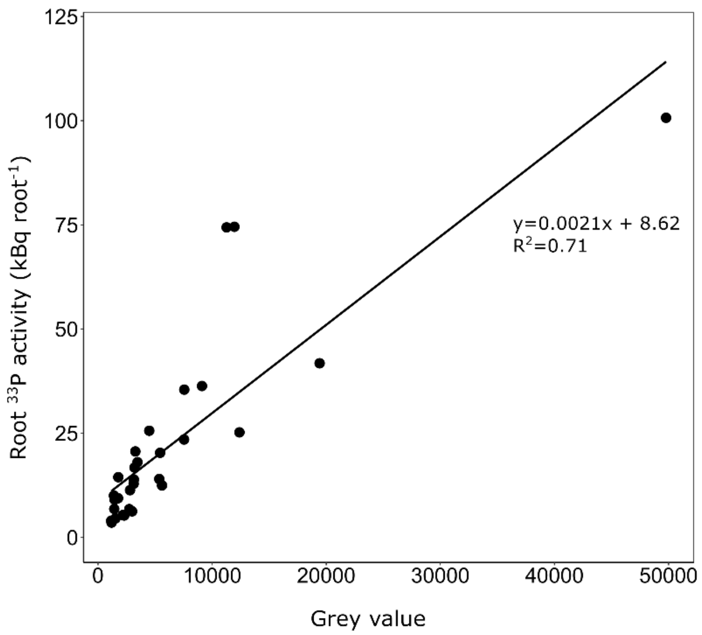

Figure S1: Correlation between grey values that were obtained after imaging the excavated root sections and the ^{33}P activity of the respective root sections in kBq per root section.

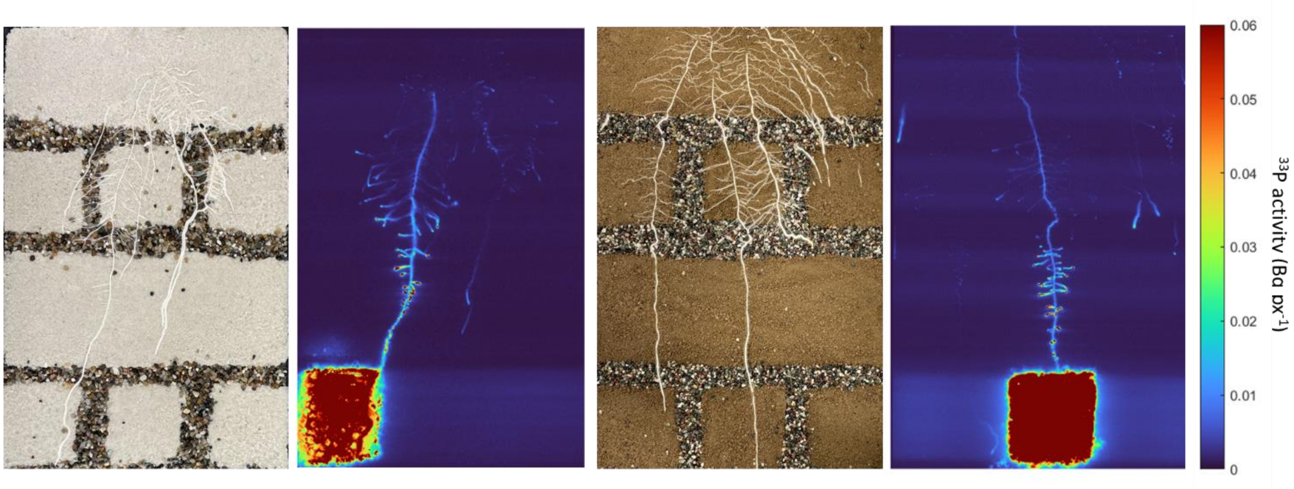

Figure S2: Two Exemplary images of one rhizobox (left) and the corresponding phosphor images taken and exemplary shown for the measurement 24 hours after labelling. The legend to the right refers to both images, the one taken from quartz sand (left) and the one taken from the sandy soil (right).

Supplementary Video: Images sequence over 7.5 hours of one rhizobox. Activity is shown in Pq px^{-1}.
